# Assessing the relevance of indicators in tracking social determinants and progress toward equitable population health in Brazil

**DOI:** 10.3402/gha.v9.29042

**Published:** 2016-02-05

**Authors:** Davide Rasella, Daiane Borges Machado, Marcelo Eduardo Pfeirrer Castellanos, Jairnilson Paim, Celia Landmann Szwarcwald, Diana Lima, Laio Magno, Leo Pedrana, Maria Guadalupe Medina, Gerson Oliveira Penna, Mauricio Lima Barreto

**Affiliations:** 1 Instituto de Saúde Coletiva, Federal University of Bahia, Salvador, Brazil; 2 Fundação Oswaldo Cruz (Fiocruz), Brasilia, Brazil; 3 ICICT, Fundação Oswaldo Cruz (Fiocruz), Rio de Janeiro, Brazil; 4 Núcleo de Medicina Tropical, University of Brasilia, Brasilia, Brazil; 5 Centro de Pesquisas Gonçalo Muniz, Fundação Oswaldo Cruz (Fiocruz), Salvador, Brazil

**Keywords:** monitoring, social determinants of health, inequality, equity, universal healthcare coverage

## Abstract

**Background:**

The importance of the social determinants of health (SDH) and barriers to the access and utilization of healthcare have been widely recognized but not previously studied in the context of universal healthcare coverage (UHC) in Brazil and other developing countries.

**Objective:**

To evaluate a set of proposed indicators of SDH and barriers to the access and utilization of healthcare – proposed by the SDH unit of the World Health Organization – with respect to their relevance in tracking progress in moving toward equitable population health and UHC in Brazil.

**Design:**

This study had a mixed methodology, combining a quantitative analysis of secondary data from governmental sources with a qualitative study comprising two focus group discussions and six key informant interviews. The set of indicators tested covered a broad range of dimensions classified by three different domains: environment quality; accountability and inclusion; and livelihood and skills. Indicators were stratified according to income quintiles, urbanization, race, and geographical region.

**Results:**

Overall, the indicators were adequate for tracking progress in terms of the SDH, equity, gender, and human rights in Brazil. Stratifications showed inequalities. The qualitative analysis revealed that many of the indicators were well known and already used by policymakers and health sector managers, whereas others were considered less useful in the Brazilian context.

**Conclusions:**

Monitoring and evaluation practices have been developed in Brazil, and the set of indicators assessed in this study could further improve these practices, especially from a health equity perspective. Socioeconomic inequalities have been reduced in Brazil in the last decade, but there is still much work to be done in relation to addressing the SDH.

## Introduction

Evidence of the importance of socioeconomic status as a determinant of population health has been growing in recent years (1), highlighting the need to include the social determinants of health (SDH) in public health policymaking (2).

Brazil is a middle-income country which has undergone substantial improvements in terms of socioeconomic conditions and health in the last decade (3, 4). Per capita income increased on average by 36.0% from 2002 to 2012 with major benefits realized by those with low education. Unemployment rates also fell in this period. In 2008, Brazil met the Millennium Development Goal on poverty reduction, but poverty remains high in some rural areas and city slums (3). Income inequality in Brazil – among the highest in the world – has also decreased (4). Average educational levels in the population have improved, and there has been a remarkable decrease in illiteracy rates (5).

Health indicators have improved in Brazil in the last decade, with the greatest reductions being in morbidity and mortality from infectious diseases in all age groups (6) but also specifically in children (7). These trends are accompanied by an increase in life expectancy (8). The rapid ageing of the population in Brazil is creating new challenges for the healthcare system, one of which is the increasing burden resulting from non-communicable chronic diseases (9).

These remarkable improvements in health conditions in Brazil have been attributed to several factors, including economic growth, increased minimum wages, and effective social and health policies (6, 7, 10). In general, there has been an expansion of the unified health system (Sistema Único de Saúde or SUS) and improvements in equity of access to healthcare, mainly in primary care (11). Although the importance of the SDH and barriers to access and utilization of healthcare have been recognized worldwide (2), these factors have not been well researched in Brazil or other developing countries moving toward universal healthcare coverage (UHC) (11). It is worth noting that in Brazil, health is the constitutional right of all citizens and the provision of healthcare is a State duty. The SUS is publicly operated and free of charge to users (12).

**Fig. 1:**
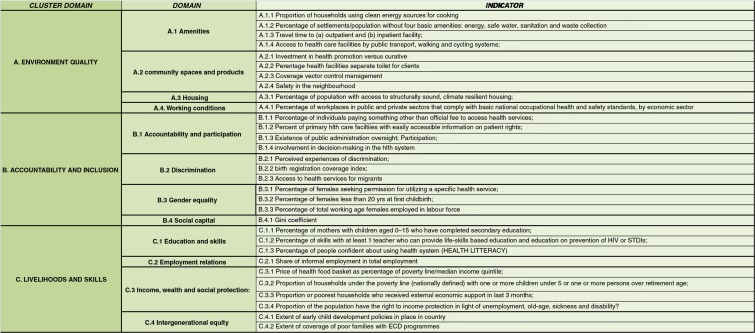
Indicators and cluster domains according to the EQuAL framework. Source: Elaboration from (unpublished result).

Ongoing monitoring of actors and conditions that have an impact on the distribution of health in a population is needed in all countries (13). The importance of data gathering and monitoring has also been highlighted in the sustainable development goals (SDGs) (14). In order to monitor intersectoral factors which influence equity-oriented progress toward achieving UHC using indicators that are comparable and relevant between and within countries, the SDH unit of the World Health Organization (WHO) has proposed a set of agreed indicators as shown in Fig. 1 (unpublished result). The indicators, which cover a broad range of SDH dimensions, are classified into three cluster domains – environment quality; accountability and inclusion; and livelihood and skills – and 12 domains, falling under the WHO equity-oriented analysis of linkages between health and other sectors (EQuAL) framework (unpublished result). The objective of this study in Brazil was to assess these indicators with respect to their relevance in tracking progress toward equitable population health and UHC.

## Methods

This study has a mixed methodology, combining a quantitative analysis of secondary data from official sources with a qualitative study comprising two focus group discussions (FGDs) and six key informant interviews (KIIs).

For the quantitative analysis, the available data – both microdata at the individual-level and/or household-level and aggregate-level data – were collected from different sources, these being the National Household Sample Survey (PNAD), the Health Supplement of the PNAD, the National Demographic Census, the National Health Survey, the National Program for Access and Quality Improvement in Primary Care (PMAQ), the World Health Survey (WHS), and the Matrix of Social Information of the Ministry of Social Development (15–21). Some indicators are reported in the Discussion section because they were only available in published studies (22–24). The analysis of microdata took into account the complex sampling design of each specific survey. Summary measures of the distribution of the indicators, including means and percentages, were calculated. In order to evaluate inequalities, indicators were stratified, where possible, according to income quintiles, urbanization, race, and geographical regions. Some double stratifications were also performed. Specific measures of inequality such as rate differences, rate ratios, and the concentration index were also used (25). Where indicators were not available, proxy indicators were used. Analyses were performed in STATA Version 12.

For the qualitative analysis, a brief summary of the main points that emerged from the FGDs and KIIs is presented. A more detailed description of the results – in a country-comparative perspective – is presented elsewhere (26). The main objective of the qualitative component was to evaluate the policy and programmatic feasibility and relevance of the indicators in terms of being understood and communicated by the target audience, and being useful within the specific policy context. Participants in the qualitative study were mainly from the health sector.

Two sets of FGDs of about 1.5 h duration were led by a coordinator and an observer. Participants were executives of the Bahia State, one of Brazil's largest states, and occupied strategic leadership positions. The initial FGD was conducted with six policymakers from different sectors who were responsible for the formulation and implementation of sectoral and intersectoral policies. Participants discussed the indicators in the domains of income and poverty, social protection, and discrimination. Participants in the second FGD were health sector managers. They discussed the indicators as a whole, covering the domains of inclusion and accountability, knowledge, livelihood/social support, and material circumstances. The FGDs allowed in-depth discussion and critique about the relevance of indicators for the orientation of public policies and programs.

KIIs of 1.5–3 h in duration were also conducted. The discussions covered the full set of indicators. Four interviews were conducted with a policymaker, a senior health sector manager, a civil society leader, and a media professional. For logistical reasons, two telephonic KIIs and two face-to-face KIIs were conducted. Key informants occupied prominent places in the government and non-government sectors at the national level and had a wealth of experience in relation to health policy and the organization of the health system.

## Results

### Quantitative analysis

In Brazil in 2012, among households under the poverty line (140 BRL per capita per month), 36.0% had at least one child under age 5 and 4.7% had a member older than 64 years, compared with 23.9% and 38.2%, respectively, in other households. The Gini index was higher in municipalities in the poorest income quintile (54.5) and lower in those in the richest quintile (48.6) (Table 1). When the municipalities were classified based on urbanization, the Gini index was lower in urban areas (49.0) than rural areas (51.6) (Table 2). The percentage of mothers with children aged 0–15 years with secondary education was 35.1%, with a large difference between the lower income quintile (11.7%) and the higher income quintile (68.1%), and a concentration index of 0.3. Almost all the houses (98.6%) used clean energy sources for cooking, such as gas and electricity, but this percentage was lower in the poorest quintile (86.6%) compared with the richest quintile (98.6%) (Table 1). Among the Brazilian households, 5.3% were without piped water, 35.1% had inadequate sanitation, 11.2% had no garbage collection, and 0.5% had no electricity in the house. When these indicators were stratified by income quintiles, strong socioeconomic gradients were evident (Table 1).

**Table 1:** Indicators according to income quintiles (first the poorest, fifth the richest), rate differences, rate ratios and concentration index

	Income quintiles						Diff. First to fifth	Ratio First/Fifth	Concentration index First to fifth
	
	First	Second	Third	Fourth	Fifth	Total			
Environment quality: amenities, community, housing									
% of hh using clean energy for cooking	88.4	95.3	95.9	98.3	99.4	95.7	−11.0	0.9	0.02
% hh without piped water	15.1	5.8	4.5	1.8	0.5	5.3	14.6	28.6	−0.48
% households without toilet	8.5	2.4	2.0	0.6	0.2	2.6	8.3	52.1	−0.54
% hh with inadequate sanitation	57.7	42.1	36.7	27.9	17.7	35.1	39.9	3.3	−0.21
% hh without garbage collection	27.5	12.8	10.8	5.1	2.2	11.2	25.4	12.8	−0.40
% hh without electricity	1.5	0.5	0.4	0.2	0.0	0.5	1.5	–	−0.51
% PHC users distant >1 km from health facility	42.4	37.8	37.8	36.5	36.9	38.3	5.5	1.1	−0.03
Mean time to reach health facility (in min)	59.1	51	38.1	39.9	38.1	45.4	21.0	1.6	−0.09
Accountability and inclusion: social capital and discrimination									
Municipal Gini index	53.8	51.0	48.1	46.0	47.7	49.3	6.2	1.1	−0.03
% PHC users who received reply to complaint	58.7	60.4	60.8	63.5	68.2	62.1	−9.4	0.9	0.03
% of population perceiving discrimination	17.1	21.2	19.5	12.6	11.4	15.9	5.7	1.5	−0.10
% mothers under 20 years	54.1	49.6	45.9	43.3	45.6	48.7	8.5	1.2	−0.04
Livelihood and skills: education, income									
% of mothers with children aged 0–15 completing secondary education	11.7	21.8	33.8	48.8	68.1	35.1	−56.3	0.2	0.30
Bolsa Familia Program coverage (%)	126.4	122.6	106.8	95.9	83.1	107.0	43.3	1.5	−0.10

Hh, household; PHC, primary healthcare. All possible stratifications of the available indicators have been included in the table. When an indicator is not stratified according to a specific stratifier is because this information was not available. Source: National Household Sample Survey (PNAD), Health Supplement of the National Household Sample Survey (PNAD), National Demographic Census, National Health Survey, National Program for Access and Quality Improvement in Primary Care, Mortality Information System, World Health Survey (2003).

**Table 2:** Indicators according to urbanization

	Urban	Rural	Total	Diff	Ratio
Municipal Gini index	47.9	50.7	49.3	−2.9	0.9
% of PHC users distant >1 km from health facility	36.2	48.7	39.74	−12.5	0.7
Mean time taken to reach health facility (in min)	41.1	63.7	45.4	−22.6	0.6
% Coverage of birth registration	98.5	96.7	97.2	1.8	1.0
% Population perceiving discrimination in healthcare facility	16.4	14.8	15.9	1.6	1.1
Bolsa Familia Program coverage (%)	110.8	103.1	107.0	−7.8	0.9

PHC, primary healthcare. All possible stratifications of the available indicators have been included in the table. When an indicator is not stratified according to a specific stratifier is because this information was not available. Source: National Household Sample Survey (PNAD), Health Supplement of the National Household Sample Survey (PNAD), National Demographic Census, National Health Survey, National Program for Access and Quality Improvement in Primary Care, Mortality Information System, World Health Survey (2003).

According to the WHS, conducted in 2003 in Brazil, the mean travel time to reach a hospital (secondary care) was 45.4 min, being 59.1 min for patients in the poorest income quintile and 38.1 min for those in the richest income quintile, 63.7 min for residents of rural municipalities and 41.1 min for urban residents (Tables 1 and 2). According to the PMAQ, in 2012, 38.4% of the users were more than 1 km away from a primary healthcare facility, this being 42.5% for users in the poorest income quintile. In Brazil in 2012, the informal employment rate was 40.5%, reaching 58.5% in the Northeast region and 30.6% in the Southeast region. About 48.7% of the mothers at their first childbirth were under 20 years.

The PMAQ for 2012 showed that 62.1% of primary healthcare users who submitted a complaint procedure to a health facility, obtained a formal reply. This percentage was higher in the richest income quintile which had a greater proportion of people with higher educational levels.

**Fig. 2:**
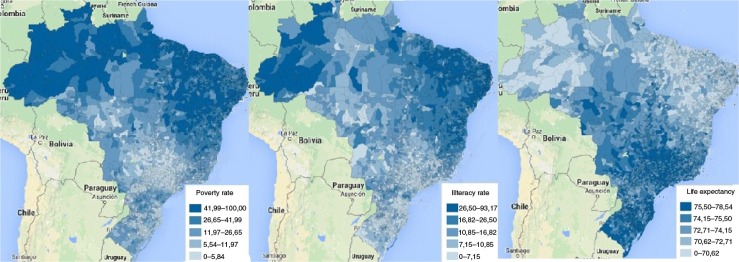
Geographical patterns of poverty rate, illiteracy rate, and life expectancy in the year 2010 in Brazil. Source: Elaboration from the Brazilian Institute of Geography and Statistics (IBGE).

In Brazil in 2010, the coverage of birth registration was 98.1%, being lower in the North region (94.9%) and in rural areas (96.7%) and higher in the Southeast region (99.0%). According to the WHS, 15.9% of the population perceived some discrimination in using healthcare services, and this was higher among females (16.5%), in the two poorest income quintiles (17.1 and 21.2%), and for younger users (18–29 years, 18.6%). Figure 2 shows the 2010 geographical patterns in poverty and illiteracy rates in Brazil. These are higher in the North and Northeast regions where life expectancies are lower. Inequalities are also evident when the same indicators are stratified according to administrative regions (Table 3), race (Table 4), and double stratification with urbanization and income quintiles (Table 5).

### Qualitative analysis

#### Focus group discussions with policymakers

In relation to the income and poverty domain, the view expressed in the FGDs was that the indicator ‘proportion of households under poverty line with one or more persons over retirement age/with one or more children under 5 has great relevance and policy and programmatic viability, being useful for intersectoral policies, especially with regard to the elderly population. The participants suggested that families with individuals with physical and/or mental handicaps should not be overlooked as vulnerable families. The Gini coefficient was regarded as having clarity and high feasibility, but participants claimed that it has low political and programmatic relevance in small areas. With regard to the social protection and employment domain, the indicator ‘share of informal employment in total employment’ was perceived as being highly communicable, but impractical. One reason for this was that there are no available reliable data on informal employment; however in general, there was controversy about the relevance of this indicator. Some participants pointed out that, especially in rural areas, there are situations where informal work is more profitable and sustainable over time than formal work. Participants understood that there was great political and programmatic relevance and viability for poverty-reduction policies involving social cash transfer policies, such as the Bolsa Familia Program, for which data are available. There was consensus about the policy and programmatic relevance of the discrimination domain.

**Table 3:** Indicators according to Brazilian administrative region

	Northeast	North	South	Central-West	Southeast	Brazil
Informal employment rate	58.5	58	31.6	36.3	30.6	40.5
% Coverage of birth registration	98.0	94.9	99.2	97.7	99.0	98.1
% Poor hh with at least one under-5	37.1	46.2	29.0	31.1	30.7	36.0
% Poor hh with at least one over 64	3.2	5.2	5.6	7.2	7.4	4.7

hh, household. All possible stratifications of the available indicators have been included in the table. When an indicator is not stratified according to a specific stratifier is because this information was not available. Source: National Household Sample Survey (PNAD), Health Supplement of the National Household Sample Survey (PNAD), National Demographic Census, National Health Survey, National Program for Access and Quality Improvement in Primary Care, Mortality Information System, World Health Survey (2003).

#### Key informant interviews

All key informants felt that the indicators concerning income and poverty, such as knowledge and education, home and infrastructure, had high relevance and policy and programmatic feasibility. In addition, all key informants perceived that ‘discrimination’ had high relevance and policy and programmatic feasibility. However, their views differed somewhat for the other domains. For the areas of community and infrastructure, responses varied between low and medium relevance and viability; social protection and employment ranged between high and medium relevance and viability; and early childhood development, ranged from high to low relevance and viability. All discussants considered gender indicators highly relevant but views on viability varied and opinions diverged regarding transparency.

#### Focus group discussions with health sector managers

Health sector managers expressed the view that, as a whole, all indicators had high programmatic relevance. However, there were some concerns about technical feasibility, with regard to the existence of reliable and affordable sources of information. There was consensus in FGDs that this set of indicators could promote the planning and evaluation of intersectoral interventions, supporting bringing health into the public policy agenda. Discussants saw the indicators as allowing the identification of vulnerable populations, often with barriers faced in accessing social protection systems, such as maternity leave, unemployment insurance, retirement pay, and pensions. They offer the potential to make an important, strong, and consistent contribution to the dialogue and the planning of intersectoral actions. Moreover, these indicators were considered relevant for assessing the impact of health programs and intersectoral actions on vulnerable populations.

**Table 4:** Indicators according to race

	Indigenous	White	Black	Yellow	Brown
% mothers with children aged 0–15 years completing secondary education	21.8	42.2	33.5	55.0	28.3
% Feeling of unsafe during the night in neighbourhood	–	56.2	55.0	–	62.4
% mothers under 20 years	63.3	50.4	42.9	40.6	47.9

All possible stratifications of the available indicators have been included in the table. When an indicator is not stratified according to a specific stratifier is because this information was not available. Source: National Household Sample Survey (PNAD), Health Supplement of the National Household Sample Survey (PNAD), National Demographic Census, National Health Survey, National Program for Access and Quality Improvement in Primary Care, Mortality Information System, World Health Survey (2003).

## Discussion

### Utility, feasibility, and relevance of the indicators

The set of indicators tested in this study are highly relevant for tracking progress toward UHC in Brazil within the context of the SDH, equity, gender, and human rights. In the quantitative analysis, all the indicators for which data were available showed some inequality when stratified. In particular, this was the case when household living conditions (absence of piped water, toilet, and electricity, among others) were stratified by income quintiles. There were also gradients and differences between population subgroups, based on geographic region and race.

In terms of technical feasibility – by which we mean ease of acquiring, analyzing and interpreting the data – the majority of the indicators were collected regularly through reliable surveys. In terms of policy and programmatic feasibility – as shown by the results of the qualitative approach – the majority of the indicators were communicable and comprehensible for policy-makers and sector managers. Overall, the indicators were considered to have high political and programmatic relevance.

### The indicators in Brazilian context

Brazil is a country where – even if great improvements in terms of socioeconomic conditions have been reached – there are still problems with regard to poverty, gender equality, and equity of access to healthcare (3, 4, 11).

Travel time and geographical distance are significant barriers to accessing healthcare facilities in developing countries (27), and in poor rural areas in these countries, transportation costs are a major inhibitor (28). In Brazil today, travel time is of relevance when considered in relation to access to hospitals and healthcare facilities that are commonly situated in urban centers (12, 29). The recent efforts of the Family Health Program (FHP) strategy to increase the coverage of primary healthcare was a welcome and important step in reducing access barriers for primary healthcare (30). The initiative has achieved important results in terms of reducing unattended child deaths and improving the collection of vital statistics and health information (31).

**Table 5:** Double stratification of indicators according to income quintiles (first the poorest, fifth the richest) and urbanization

	First		Second		Third		Fourth		Fifth	
					
	Urban	Rural	Urban	Rural	Urban	Rural	Urban	Rural	Urban	Rural
Municipal Gini index	51.8	54.1	50.4	51.3	47.5	48.7	45.7	46.7	48.4	45.5
(%) Bolsa Familia Program coverage	126.3	126.4	125.1	121.2	110.9	102.1	98.8	88.3	87.6	68.7

All possible stratifications of the available indicators have been included in the table. When an indicator is not stratified according to a specific stratifier is because this information was not available. Source: National Household Sample Survey (PNAD), Health Supplement of the National Household Sample Survey (PNAD), National Demographic Census, National Health Survey, National Program for Access and Quality Improvement in Primary Care, Mortality Information System, World Health Survey (2003).

Discrimination is an issue in Brazil (32, 33). This occurs in relation to income and socioeconomic status as shown in WHS data (20). Brazilian women are also more susceptible to discrimination if they are black, unmarried, have a low income, or are pregnant and adolescent (34, 35). However, progress has been made in improving gender equality. The gender gap index, which is an international measure of gender equality, improved from 0.654 to 0.695 in Brazil during the period 2006–2011, ranking the country in 62th position in the world. However, Brazil does not rate high when compared with some other developing countries (36). In Brazil, women have more access to healthcare than men, and are not judged by men for seeking healthcare (37). They seek more preventive care than men, in particular for routine examinations and curative care (38). Concerning early child development, in 2001 laws were introduced to protect the health and rights of children and adolescents (39). The Brazilian government has also implemented programs, such as the National Policy of Alimentation and Nutrition, and the Milk Program for Life, the main objective of which is to reduce the malnutrition and child mortality (40).

In the last two decades, the Brazilian public health system increased investment in health promotion and prevention (41), but, as is the case in other countries, it is difficult to quantify the amount of money invested because health promotion/prevention and curative budgets are often combined (42, 43). The Brazilian unified health system (SUS) is still based on curative care. With rapid population ageing, there needs to be more investment in preventive care (9, 44, 45).

The involvement of the population in the decision making process for health was institutionalized in Brazil in 1988. This involved the establishment of the ‘Conselhos de Saude’ (CS), permanent commissions composed by representatives of government, of other non-governmental health providers, health professionals, and users of the health system (12). However, the CS faces many challenge in ensuring the smooth running and consolidation of processes (46).

Informal workers in developing countries are usually strongly disadvantaged due to the lack of protection and in Brazil work in the private sector, businesses, and domestic services (22, 47).

Corruption, at different levels, is still a major problem, with a Corruption Perception Index of 42 in 2012 (48, 49). During the last decade, several laws have been implemented to increase accountability and transparency in the management of public funds, including the compulsory provision of budget information in internet (50). Violence represents one important cause of death and of hospital admissions in Brazil (51). Homicide alone accounts for 36.4% of all deaths from external causes and has major negative social impacts in communities throughout the population (52).

The main limitation of this study was that we used and assessed a high number of wide ranging indicators. This limited the possibility of analyzing individual indicators in any depth, either in the quantitative or qualitative analysis. This study has many strengths. To our knowledge, this is the first study of its kind. The setting is important because Brazil is an information-rich country, where most of the indicators of the EQuAL framework are already or can potentially be collected.

The importance of monitoring population conditions in developing countries has been recently highlighted in the 2030 Agenda for Sustainable Development, with two SDGs (17.18 and 17.19) dedicated to data, monitoring, and accountability (14). Data on the SDH should be gathered to not only identify inequalities under an equity focus (13), but also in order to understand how health outcomes are or will be played out under future scenarios such as UHC. The variety of domains for SDH monitoring, such as the EQuAL framework, highlights the need for an interdisciplinary approach, both at academic and policymaking levels (2, 53, 54).

## Conclusions

In Brazil, inequalities among the population are still present and the monitoring of the SDH and barriers to healthcare is an essential step toward achieving UHC in the context of the country's unified health system.

In order to be useful for policymakers and health sector managers, the indicators from the different domains need to be reliable, valid, feasible, and relevant, as well as up-to-date. Most of the indicators discussed here in the context of Brazil have these characteristics. As shown by recent studies, intersectoral policies which address the SDH can be extremely effective in reducing health inequalities (10, 55). Great advances have been achieved in Brazil in the last decade, but large inequities still exist. Public policies should be focused on achieving a more equitable distribution of health as the country moves toward UHC.

## Ethics and consent

No ethical approval was necessary for this study. The quantitative data were analyzed in aggregated de-identified format. In the qualitative component, all discussions were of a professional nature and were conducted in workplace settings.
